# Supplementary material for: Antifungal activity and mechanism of novel peptide Glycine max antimicrobial peptide (GmAMP) against fluconazole-resistant Candida tropicalis
Source: PeerJ. 2025 May 20;13:e19372. doi: 10.7717/peerj.19372 (PMC12101439; doi:10.7717/peerj.19372)
Supplement: Supplemental Information 7 — (A) purification of GmAMP. (B) electrospray ionization (ESI) mass spectrometer of GmAMP. [file peerj-13-19372-s007.docx]

**A**

Peak No. Time Area Height Conc.

1 7.500 23337 982 0.9846

2 8.045 39420 1648 1.6630

3 8.422 2259521 217332 95.3300

4 8.780 25982 6922 1.0960

5 17.780 21872 2502 0.9229

Total 100.0000

Column :4.6x250mm C18

Solvent A :0.1% Trifluoroacetic in 100% Acetonitrile

Solvent B :0.1% Trifluoroacetic in 100% Water

Gradient: A B

0.01min 10% 90%

25min 35% 65%

25.01min 100% 0%

30.0min STOP

Flow rate :1.0 ml/min

Wavelength :220nm

Volume :10µl

|   **B**  M.W.: 2539.35  Instrument Agilent-6125B  Probe: ESI Probe Bias: ＋4.5kv  Nebulizer Gas Flow: 1.5L/min Detector: 1.5kv  CDL: -20.0v T. Flow: 0.2ml/min  CDL Temp.: 250 ℃ B. Conc.: 50%H2O/50%ACN  Block Temp.: 200℃  [M+6H]6+  [M+3H]3+  [M+4H]4+  [M+5H]5+ |
| --- |

**Figure. S. 1.** **Characterization of GmAMP.** (A) purification of GmAMP. (B) electrospray ionization (ESI) mass spectrometer of GmAMP.
